# Reply to Rakovich *et al.*

**DOI:** 10.1093/icvts/ivac110

**Published:** 2022-07-25

**Authors:** Natasha Toleska Dimitrovska, Xiao Chu, Wentao Li

**Affiliations:** Department of Thoracic Surgery, University Clinic for Thoracic and Vascular Surgery, Skopje, The Former Republic of Yugoslavia; Department of Thoracic Surgery, The Fifth People’s Hospital of Shanghai, Fudan University, Shanghai, China; Department of Thoracic Surgery, Shanghai Jiao Tong University Affiliated Chest Hospital, Shanghai, China

**Keywords:** Learning curve, Segmentectomy, Video-assisted thoracoscopic surgery

We thank Dimitrovska *et al.* for their interest in our work [1]. We have read the comments carefully and enclosed are my replies to some of their questions and suggestions.

First, Woodall *et al.* proposed possible problems with 3-stage classification [2]. Although it has several limitations, the cumulative sum (CUSUM) analysis which divide the learning process into three stages was the most commonly used tool for quantitative assessment of a learning curve. One of these studies is titled ‘Learning curve and associated morbidity of minimally invasive oesophagectomy: a retrospective multicentre study’ published by van Workum *et al* [3]. Another of these studies is ‘Learning curve of robot-assisted transabdominal preperitoneal (rTAPP) inguinal hernia repair: a cumulative sum (CUSUM) analysis’ by Kudsi *et al.* [4].

We conducted the following analysis to assess the rationality of the 3-stage classification that they were critical of in the letter. The mean (± standard deviation) CUSUM value in the 3 phases was 329.77 ± 182.76, 641.49 ± 18.81 and 348.91 ± 189.40, respectively (Table 1). The standard deviation of the CUSUM value was smaller in phase II than in phases I and III. This was the rationale to divide the learning curve into 3 phases.

**Table 1:** Variation in the CUSUM value in the 3 phases

	Phase I	Phase II	Phase III	*P*-value
CUSUM value, mean ± SD	329.77 ± 182.76	641.49 ± 18.81	348.91 ± 189.40	<0.001
CUSUM value, median (IQR)	450.34 (355.51–557.16)	639.46 (628.91–657.29)	327.48 (168.20–491.76)	<0.001

CUSUM: cumulative sum; SD: standard deviation; IQR: interquartile range.

Considering their proposition that the average duration of surgery presents a linear trend with decrease in surgical duration, we introduced a simple linear regression model to explore the change in learning time while considering the variables in Table 1 of our original paper to observe their impact on the surgical duration. The final coefficient of determination of the linear regression model was small (*R*^2^ = 0.420 after adjustment), indicating that only 42.0% of the change in surgical duration could be explained by this model. By contrast, the coefficient of determination (*R*^2^ = 0.894) of the learning time curve fitted with the CUSUM analysis was greater and is thus an improvement to the simple linear regression model. In addition, the linear regression model requires the independence of observed objects [5]. For the same doctor, the surgical duration will definitely decrease with an increase in surgical proficiency. In other words, surgical duration is inevitably affected by the accumulation of experience, so the observed objects in this study are not independent.

Regarding whether stability in the surgical duration was achieved, we can see from the CUSUM value that in the final stage, the duration of surgery began to decrease after the doctors had become fully familiar with the procedure. There was no difference between the duration of the 86th procedure and the mean surgical duration, proving that after 86 procedures, the duration of surgery was stable.

Finally, they mentioned that adding any constant (like grouping by 30) to the surgical duration would not affect the CUSUM value. An increase in the constant would only affect the mean value and not the variability. We agree with them in this respect. However, this property of the CUSUM analysis does not affect the results and conclusions of this study.
